# PRR2, a pseudo-response regulator, promotes salicylic acid and camalexin accumulation during plant immunity

**DOI:** 10.1038/s41598-017-07535-8

**Published:** 2017-08-01

**Authors:** C. Cheval, M. Perez, L. J. Leba, B. Ranty, A. Perochon, M. Reichelt, A. Mithöfer, E. Robe, C. Mazars, J. P. Galaud, D. Aldon

**Affiliations:** 1 Laboratoire de Recherche en Sciences Végétales, Université de Toulouse, CNRS, UPS, 24 chemin de Borde Rouge, Auzeville, BP42617, 31326 Castanet-Tolosan, France; 2 Department of Biochemistry, Max Planck Institute for Chemical Ecology, Hans Knöll Strasse 8, 07745 Jena, Germany; 3 Department of Bioorganic Chemistry, Max Planck Institute for Chemical Ecology, Hans Knöll Strasse 8, 07745 Jena, Germany; 4 Present Address: John Innes Centre, Norwich Research Park, Norwich, NR4 7UH UK; 5 UMR QualiSud, Université de Guyane, Campus Universitaire de Troubiran, P.O. Box 792, 97337 Cayenne Cedex, French Guiana France; 6 Present Address: University College Dublin Earth Institute and School of Biology and Environmental Science, College of Science, University College Dublin, Belfield, Dublin Ireland

## Abstract

Calcium signalling mediated by Calmodulin (CaM) and calmodulin-like (CML) proteins is critical to plant immunity. CaM and CML regulate a wide range of target proteins and cellular responses. While many CaM-binding proteins have been identified, few have been characterized for their specific role in plant immunity. Here, we report new data on the biological function of a CML-interacting partner, PRR2 (*PSEUDO-RESPONSE REGULATOR 2*), a plant specific transcription factor. Until now, the physiological relevance of PRR2 remained largely unknown. Using a reverse genetic strategy in *A*. *thaliana*, we identified PRR2 as a positive regulator of plant immunity. We propose that PRR2 contributes to salicylic acid (SA)-dependent responses when challenged with the phytopathogenic bacterium *Pseudomonas syringae*. *PRR2* is transcriptionally upregulated by SA and *P*. *syringae*, enhances SA biosynthesis and SA signalling responses; *e*.*g*. in response to *P*. *syringae*, PRR2 induces the production of SA and the accumulation of the defence-related protein PR1. Moreover, PRR2 overexpressing lines exhibit an enhanced production of camalexin, a phytoalexin that confers enhanced resistance against pathogens. Together, these data reveal the importance of PRR2 in plant immune responses against *P*. *syringae* and suggest a novel function for this particular plant specific transcription factor in plant physiology.

## Introduction

Plants have a great potential to adapt their growth and development to environmental changes. This phenotypic plasticity relies on the ability to simultaneously integrate a wide variety of abiotic stimuli (light, temperature, nutrients…) and biotic interactions (pathogens, symbionts and others), through a network of signalling pathways mediated by second messengers and phytohormones.

Downstream of these complex signalling networks, a multitude of transcription factors (TFs) regulate the expression of stress-responsive genes. These TFs have been associated to plant defence against pathogens or to plant adaptation in response to abiotic stresses and their abundance and specificity depends on the nature and strength of the stress challenge^1, 2^. In many cases, these TFs are regulated at both transcriptional and post-translational level such as phosphorylation and ubiquitination but also by protein-protein interactions^3–5^.

A clear link between the Ca^2+^ signalling pathway and transcriptional reprogramming is now well established. The calmodulin (CaM), an ubiquitous calcium sensor found in all eukaryotes is a central regulator of TFs dynamics. In plants, the CaM orchestrates the activity of several TFs such as CAMTAs, WRKYs and MYBs that directly interact with the CaM^6–9^.

Functional analyses of some of these CaM-interacting TFs support their roles in stress signalling pathways induced by both abiotic and biotic cues^8, 10^. For instance, the *camta3* loss-of-function mutants displayed an increase resistance to pathogens associated with elevated salicylic acid (SA) levels and enhanced expression of defence-related genes^11, 12^. On the contrary, *cbp60g* knockout mutants were shown to be defective in the accumulation of SA in response to pathogen infection^13^. These data illustrate a first level of complexity where CaM-interacting TFs play antagonistic roles in plant immunity by modulating the production of SA. Compared to other eukaryotes, plants also present a range of calmodulin-related proteins (CMLs) which interact with a broad spectrum of target proteins including many TFs^14, 15^. This considerable number of CaM/CMLs-interacting TFs creates another level of complexity. In most cases, the biological relevance of these CML-TFs interactions remains to be elucidated.

A reverse genetic approach in the plant model *Arabidopsis thaliana* using gain and loss of function transgenic lines provided evidence for a role of CML9 in abiotic stress responses^16^ but also in plant immunity^17^. The search for CML9-regulated components identified the Pseudo-Response Regulator 2, (PRR2) as a CML9-interacting protein^18^. PRR2 is an atypical pseudo-response regulator (PRRs). PRRs were reported to be associated to the two-component system^19^ and several PRRs such as TOC1 have been shown to play a key role in the circadian clock mechanism^20^, whereas very little is known about the role of other PRRs. PRR2 possesses a Myb-like DNA binding domain also referred to a GARP domain and localizes to the nucleus^18^. However, the physiological function of PRR2 still remains unknown. The data obtained on the putative tomato orthologue of PRR2, SlPRR2, indicate its contribution to fruit pigmentation and ripening^21^.

In the present study, by using knock-down mutant lines and transgenic *Arabidopsis* plants exhibiting an ectopic expression of *PRR2*, we bring evidences that PRR2 is involved in defence responses against the phytopathogenic bacteria *Pseudomonas syringae*. We show that PRR2 contributes to SA-dependent defence responses and enhances the production and the accumulation of camalexin.

## Results

### PRR2 is mainly expressed in aerial parts during plant development

**Figure 1:**
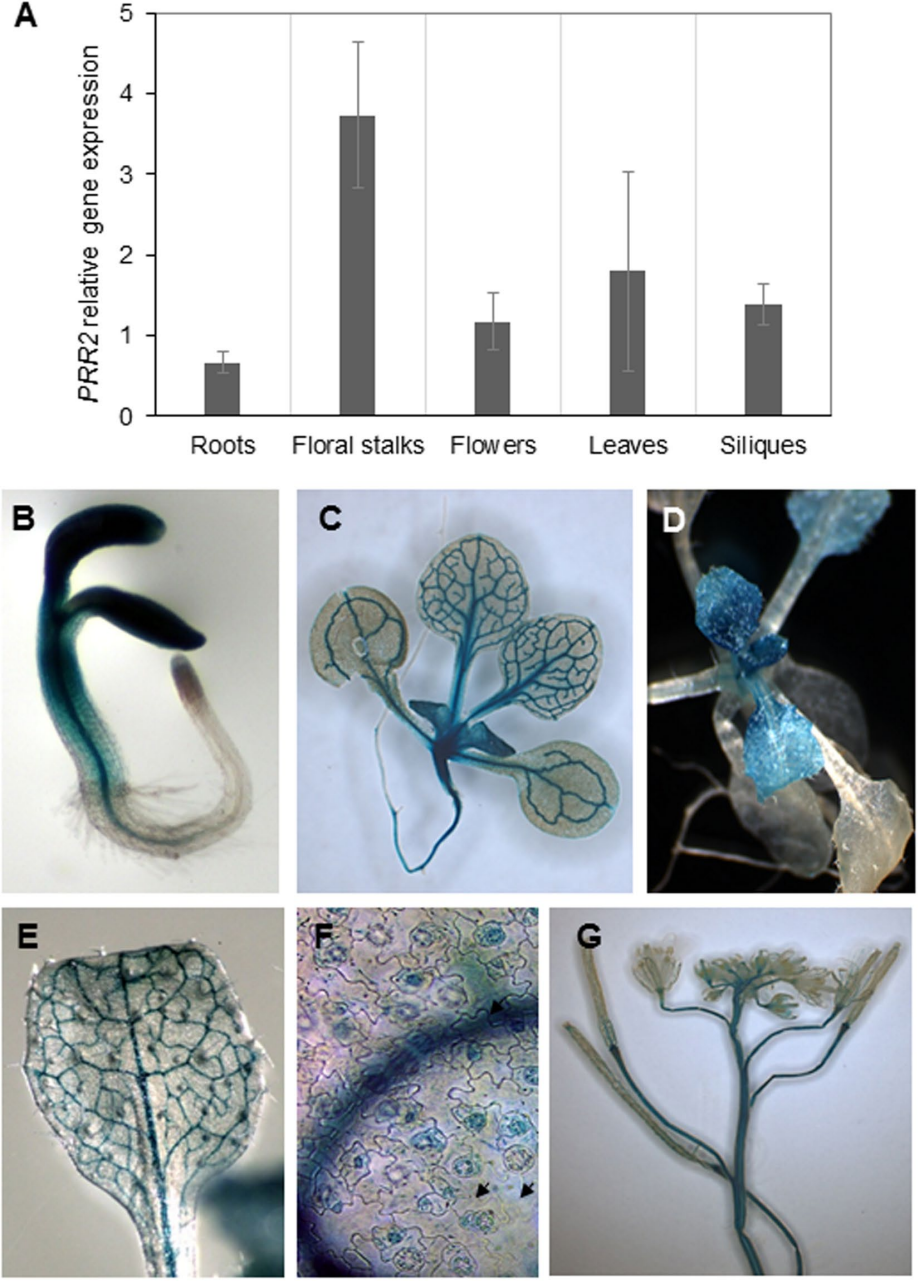
*PRR2 gene* expression patterns analyses. (**A**) RT-_Q_PCR analysis of *PRR2* transcript levels in different organs of *Arabidopsis thaliana* (Col) plants. Total RNAs purified from different tissues of plants grown under long-day conditions were subjected to RT-_Q_PCR with specific primers for *PRR2*. The data were obtained by the comparative 2^−ΔΔCT^ method using *actin8* as a reference gene. The illustrated values are means ± SD of four independent experiments. (**B** to **G**) Histochemical GUS staining in tissues of transgenic plants transformed with *PRR2* promoter::*uidA* reporter construct. (**B**) 4-day-old seedling; (**C**) 16-day-old seedling; (**D**) 3-week-old plant; (**E**) adult leaf; (**F**) leaf epidermal tissue and guard cells (arrows); (**G**) floral stalk and developing siliques. T_3_ generation plants were grown under long-day conditions and subjected to GUS staining overnight. The observations illustrated are representative of patterns obtained with five independent transgenic lines.

In order to detail *PRR2* gene expression patterns, we investigated *PRR2* gene expression at the organ level using Real-Time Quantitative Reverse Transcription PCR (RT-_Q_PCR) and its spatio- temporal regulation using transgenic plants carrying the *Pro*_*PRR2*_::*uidA* gene fusion. RT-_Q_PCR analyses indicated that *PRR2* is expressed in all major organs of adult plants, but mainly in rosette leaves, inflorescence stems, flowers, and siliques (Fig. 1A). We generated independent transgenic plants carrying the *Pro*_*PRR2*_::*uidA* gene fusion composed of a 2.3 kb region upstream of the start codon of *PRR2* coding sequence fused to the *GUS* reporter gene. GUS staining was performed on five independent lines and similar GUS staining patterns were obtained. Histochemical GUS staining revealed the localization of *PRR2* expression in tissues (Fig. 1B to G). In four-day-old seedlings, GUS activity was observed throughout the cotyledons, hypocotyls, but not in the root tip (Fig. 1B). Significant expression of *Pro*_*PRR2*_::*uidA* was detected in young leaves (Fig. 1C to E) with a strong *GUS* staining associated to the vasculature (Fig. 1C,E and G) and in epidermal specialized leaf structures such as guard cells (Fig. 1F). These data confirm the available information coming from several microarray databases concerning *PRR2* gene expression^22^. All together, these expression analyses support that PRR2 is likely to play a role in aerial parts of the plant throughout development and morphogenesis.

### PRR2 gene expression is induced in a SA-dependent manner in response to *P. syringae* (*Pst* DC3000)

**Figure 2:**
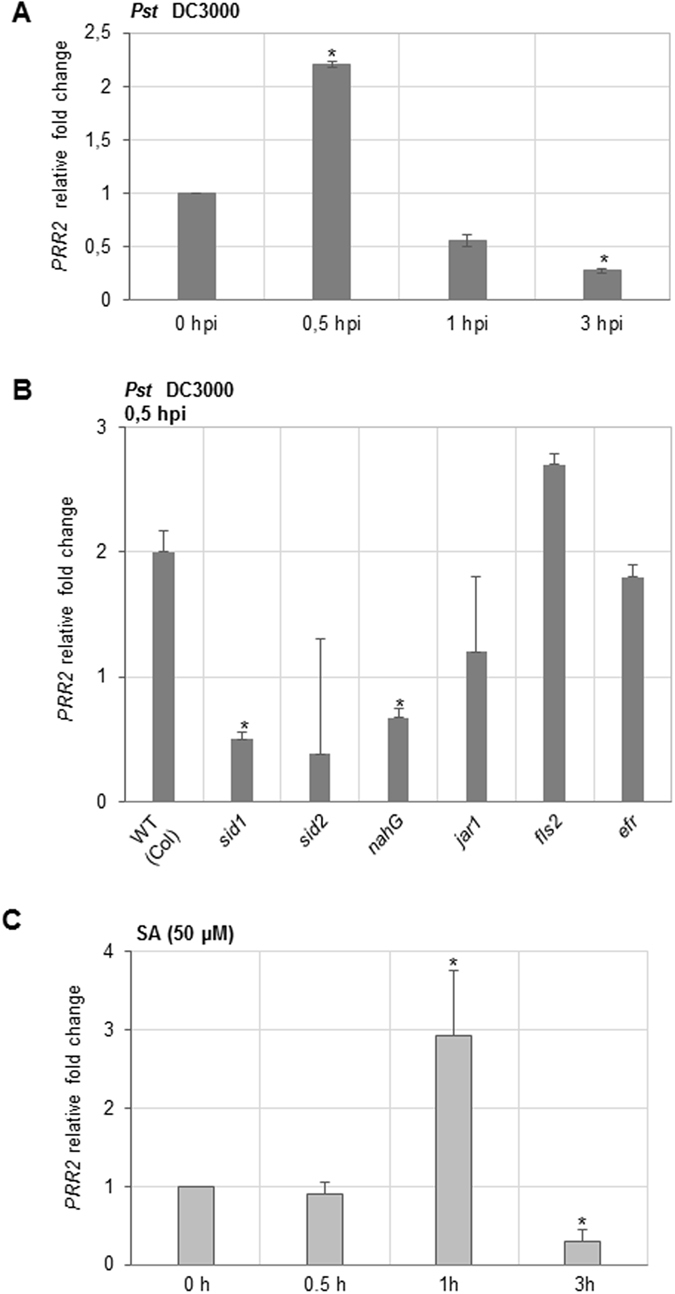
*PRR2* gene expression analyses in response to *Pseudomonas syringae* inoculation and to salicylic acid treatment. (**A**) *PRR2* gene expression in response to *P*. *syringae* infection. Leaves of 4-week-old Col plants were inoculated with *Pst* DC3000 at 5.10^7^ cfu.mL^−1^. Samples were collected at 0, 0.5, 1 and 3 h post-inoculation (hpi). (**B**) *PRR2* gene expression in mutants defective for hormonal production and MAMP perception. *PRR2* expression was monitored 0.5 h after *Pst DC3000* inoculation in 4-week-old Arabidopsis mutants defective for SA production (*sid1*, *sid2* and a line carrying the *nahG* transgene), JA (*jar1*) signalling pathways but also in mutants altered in flagellin (*fls2*) or EF-Tu (*efr*) perception. (**C**) Time-course of *PRR2* gene expression in response to exogenous application of salicylic acid (SA 50 µM). Seedlings were collected at 0, 0.5, 1 and 3 h after SA treatment. All the expression analyses are presented as a fold change relative to mock treatment. Relative transcript quantification was assayed by quantitative real-time PCR and calculated by the comparative 2^−ΔΔCT^ method using *actin8* as a reference gene. Data illustrated represent the mean ± SE of three biological replicates from two independent experiments. Asterisks (*) above histograms (ANOVA, p-value 0.05) indicate significant changes of *PRR2* gene expression in these different genetic backgrounds compared to WT (Col).

PRR2 was firstly identified as a CML9-interacting partner. We have previously demonstrated that CML9 regulates plant defence responses when challenged with *Pst* DC3000^17^. Therefore we have investigated the regulation of *PRR2* gene expression in response to this particular bacterial pathogen. We infiltrated *Arabidopsis* WT leaves (accession Col0) with the virulent *Pseudomonas syringae pv tomato* (*Pst*) strain DC3000 and *PRR2* gene expression was quantified using RT-_Q_PCR. Upon foliar inoculation, a moderate (2-fold) but significant up-regulation of *PRR2* is observed after 30 min. This induction of gene expression is transient and decreases below its basal level 1 h and 3 h post-inoculation (Fig. 2A). Since phytohormones like salicylic acid (SA) and jasmonic acid (JA) were clearly shown to orchestrate the plant defence responses to *Pst* ^23^, we investigated the regulation of *PRR2* gene expression in response to these hormonal compounds. *PRR2* gene expression level was quantified in wild-type plants (Col) and in mutants or transgenic lines altered in the production of SA (*nahG*, *sid1*, *sid2*), JA signalling (*jar1*) following inoculation with *Pst* DC3000 (Fig. 2B). In response to *Pst*, the establishment of plant defence responses relies on the perception of pathogen-associated molecular patterns (PAMPs) through pattern recognition receptors (PRRs). Therefore, we also evaluated the possible contribution of the two receptors involved in the perception of the flagellin (FLS2) and the elongation factor EF-Tu (EFR) in the regulation of *PRR2* gene expression using the corresponding *fls2* and *efr* mutant lines (Fig. 2B). RT-_Q_PCR analyses revealed that *PRR2* gene expression is not significantly modified in *jar1*, *fls2* and *efr* mutants compared to WT plants (Fig. 2B). *PRR2* gene induction is therefore independent from the JA-signalling pathway and not regulated by the functional receptors FLS2 and EFR in response to *Pst*. In contrast, data clearly demonstrated that an alteration of SA production in *nahG* transgenic line and *sid* mutants leads to a significant reduction of *PRR2* gene transcription (Fig. 2B). These results indicate that *PRR2* gene expression depends on an activated SA-signalling pathway during *A*. *thaliana*–*Pst* interaction. To support these data, the effect of an exogenous SA application (50 µM) on *PRR2* gene expression was quantified in *in vitro* grown seedlings (12 day-old plants) (Fig. 2C). In this experimental design, we previously confirmed that SA exogenous treatments induce the expression of the SA-dependent marker gene *PR1* in WT plants (data not shown). *PRR2* gene expression is transiently induced 1 h after treatment (3-fold induction) and decreases below its initial level within 3 h (Fig. 2C). These data confirm that *PRR2* gene induction is controlled by the SA-dependent signalling pathway.

### PRR2 is a positive regulator of plant defence in response to *P*. *syringae* infection

To identify the biological function of PRR2 in response to *P*. *syringae*, we carried out a reverse genetic approach *in planta*. Two homozygous *Arabidopsis* lines harboring a T-DNA insertion in *PRR2* gene (*prr2*.*1* (Col) *and prr2*.*2* (WS)) were characterized (Supplemental Informations 1 and 2). These mutants only present a down-regulation of *PRR2* gene expression (Supplemental Information 1) since no T-DNA insertion lines that lead to a complete knockout of *PRR2* were available. We generated *Arabidopsis* (Col) transgenic lines expressing the coding sequence of *PRR2* fused to a hemagglutinin (HA) tag under the control of the constitutive 35S promoter (*p35S*::*cdsPRR2-HA*). Two independent lines (*OE-PRR2*.*1* and *OE-PRR2*.*2*) displaying a constitutive and strong PRR2 expression both at the transcript and protein levels were characterized and selected (Supplemental Information 2). No morphological and developmental defects were observed in these transgenic lines compared to WT plants under normal growth conditions (Supplemental Information 3).

**Figure 3:**
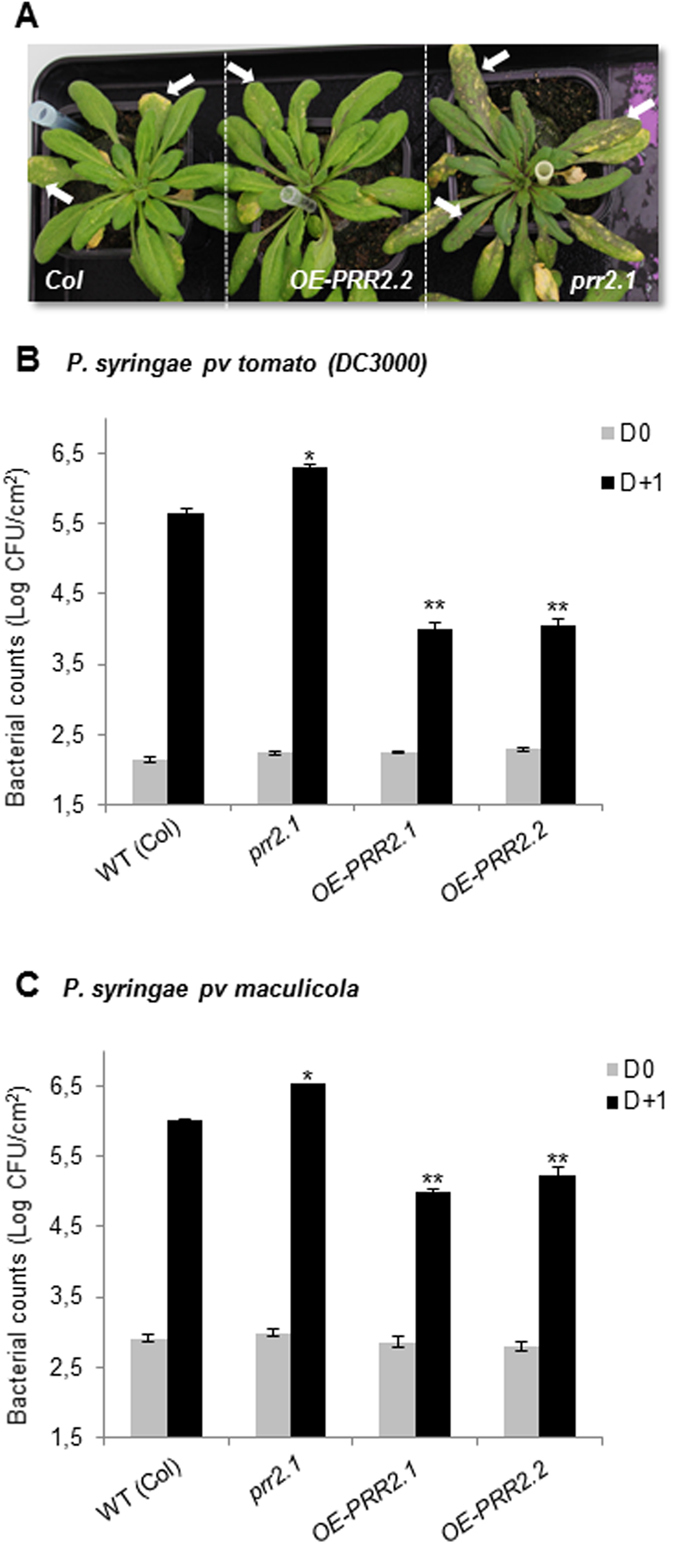
Altered susceptibility to *Pseudomonas syringae* in *prr2* knock-down mutants and in transgenic lines overexpressing *PRR2*. (**A**) Disease symptoms observed in 4-week-old *Arabidopsis* leaves (WT (Col), *OE-PRR2*.*2 and prr2*.*1*) caused by *Pst* DC3000 infection. Leaves were sprayed with 5.10^7^ cfu.mL^−1^ of *Pst* DC3000 and pictures were taken 7 days post-infection. Arrows indicate leaves exhibiting pronounced chlorosis symptoms. (**B** and **C**) Quantification of *in planta* bacterial growth were performed at 0 and 1 dpi with *Pst* DC3000 (**B**) or *Pseudomonas syringae pv maculicola* (**C**) in WT (Col), *prr2*.*1* mutant and over-expressing transgenic lines *OE-PRR2*.*1* and *OE-PRR2*.*2*. Data are representatives of 8 replicates from three independent experiments (n = 24). Error bars indicate SE. P values were calculated using the two-tailed Mann-Whitney U-test to indicate significant differences in bacterial growth in these different genetic backgrounds compared to WT (*p < 0.05, **p < 0.01).

To determine whether PRR2 plays a role in defence responses against *P*. *syringae*, we firstly examined the behavior of *prr2*.*1* mutant line and overexpressors of *PRR2* (*OE-PRR2*.*1* and *OE-PRR2*.*2*) after spray inoculation of leaves with 5 × 10^7^ cfu.mL^−1^ of the virulent strains of *Pst* DC3000 (Fig. 3A,B). Chlorosis symptoms observations after seven days clearly indicate that the *prr2*.*1* line exhibits more pronounced symptoms in comparison to the WT or *OE-PRR2*.*1* (Fig. 3A). *In planta* bacterial growth was then quantified in infected leaves and results indicate no bacterial growth difference between the genotypes just after spraying (0 dpi). However, *prr2*.*1* mutants plants display a 4-fold increase in bacterial titer compared to WT-infected plants after 1 dpi (Fig. 3B) whereas bacterial growth is 44-fold lower in the *OE-PRR2s* (Fig. 3B). Using *Pst* DC3000 syringe-infiltration method, similar results were obtained (data not shown). These experiments were also performed in the WS accession and results indicate a significant increase of *in planta* bacterial growth in *prr2*.*2* compared to WT plants after 1 dpi (Supplemental Information 4A). To support and strengthen these data, infection assays were performed using another virulent pathogen *Pseudomonas syringae pv maculicola*, a crucifer-specific bacterial strain. Results were comparable to those obtained with the DC3000 strain (Fig. 3C). Together, these pathoassays demonstrate an enhanced susceptibility of *prr2* mutants and an increased disease resistance of *OE-PRR2* lines compared to WT plants in response to *P*. *syringae* (Figs 3 and SI 4). This indicates that PRR2 contributes to disease resistance in response to *P*. *syringae* infection.

### Expression of defence-associated markers is altered in *prr2* genotypes following *P*. *syringae* inoculation

The present study indicates that *PRR2* is induced in a SA-dependent manner. To evaluate the contribution of PRR2 to SA-dependent responses, we examined several molecular and biochemical markers associated to SA in *prr2* transgenic lines. We firstly performed expression analyses of genes associated to defence responses following *Pst* infection. Only early time points after infection were analyzed as these have been found to be most significant contributors to the outcome of infection^24^. Those selected genes encode transcription factors and proteins known to be involved in phytoalexin production, SA synthesis and signalling. Gene expression was evaluated by RT-_Q_PCR using RNA extracted from WT (Col), *prr2*.*1* mutant and *OE-PRR2*.*1* plants, at different time points after inoculation with *Pst*.

**Figure 4:**
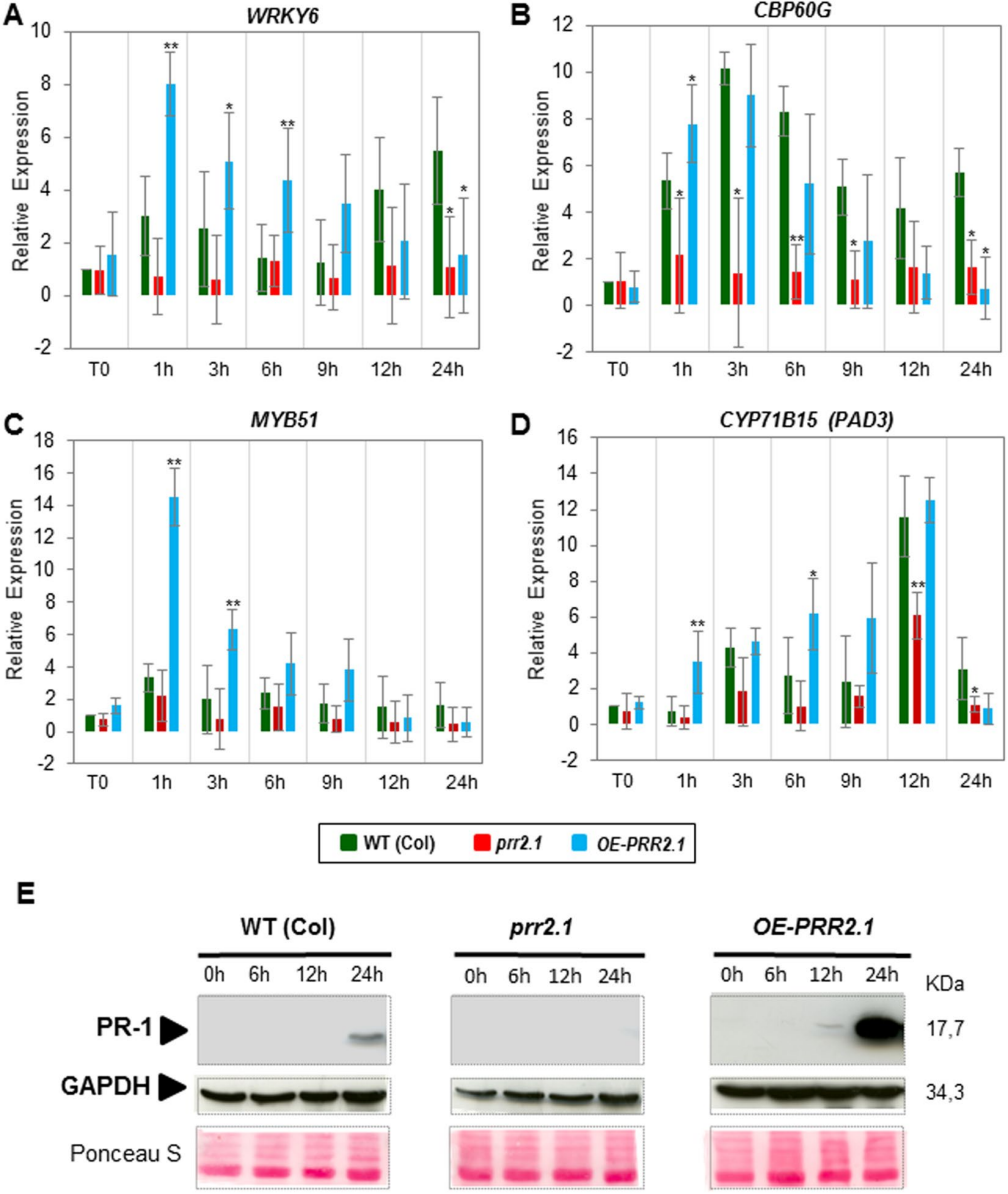
Expression analysis of defence marker genes and detection of PR1 protein in *prr2* knock-down mutants and in transgenic line overexpressing *PRR2*. (**A**,**B**,**C** and **D**) Analyses of *WRKY6* (**A**), *CBP60G* (**B**), *MYB51* (**C**), *CYP71B15/PAD3* (**D**) marker genes in different *prr2* genetic backgrounds. Leaves of 4-week-old *Arabidopsis* WT (Col), mutant *prr2*.*1*, and *OE-PRR2*.*1* were inoculated with 5 × 10^7^ cfu.mL^−1^ of *Pst* DC3000 and harvested at 0, 1, 3, 6, 9, 12 and 24 hours post-inoculation (hpi). The fold changes relative to the mock treatment were determined by RT-_Q_ PCR. The values are means ± standard deviation of three independent experiments. Asterisks (*) above histograms indicate significant changes of *PRR2* gene expression in these different genetic backgrounds compared to WT (Col) (student t-test with p-value < 0.05 (*) or p-value 0.01 (**)). (**E**) Detection of PR1 by immunoblot experiment in WT plants and *prr2* genotypes. PR1 accumulation was detected in leaves of 3-week-old plants at 0, 6, 12, 24 hpi after spraying with *Pst* DC3000 (2 × 10^8^ cfu.mL^−1^). Equal loading was confirmed by immunoblot detection of GAPDH and Ponceau S staining of the membrane (middle and lower panels). The illustrated blot is representative of three biological replicates.

The expression profiles of marker genes associated with SA (*WRKY6 and CBP60G*) and camalexin synthesis (*MYB51* and *PAD3*) are illustrated (Fig. 4A to D). In control conditions (T0), no significant difference in gene expression level is observed between genotypes for any of these genes. However, we observed significant changes in gene expression at early and/or late-time points following *Pst* infection.

In our experiments, *WRKY6*, that encodes a transcription factor involved in plant defence^25^, is induced at late time-points (12 h–24 hpi) in WT plants. In comparison, we observed a rapid (early time-points, 1 to 9 hpi) and strong induction of this gene in *OE-PRR2*.*1* lines and a significant and constant repression in *prr2*.*1* lines (Fig. 4A). CBP60g is a positive regulator of plant immunity that promotes the production of SA^13^. *CBP60g* gene induction is not significantly modified by the over-expression of *PRR2* in *OE-PRR2*.*1* lines compared to the WT whereas a significant and constant repression is observed in *prr2*.*1* lines (Fig. 4B).

We identified similar patterns of expression for genes involved in camalexin synthesis (Fig. 4C and D). *MYB51* gene expression increases strongly and significantly at early time-points (1 to 9 hpi) in *OE-PRR2*.*1* compared to WT and *prr2*.*1* (Fig. 4D). CYP71B15 (*PAD3*) gene encodes the enzyme catalyzing the final step of the camalexin biosynthetic pathway^26, 27^. The *CYP71B15* (*PAD3*) gene is rapidly, strongly and significantly expressed 1 hpi in *OE-PRR2* compared with WT and *prr2*.*1*. *CYP71B15* expression level increased with time, up to 12 hpi before going back to the basal level at 24 hpi. (Fig. 4D).

All these results indicate that PRR2 regulates gene expression associated to SA homeostasis and the production of camalexin in response to *Pst*.

To support this data, we examined the Pathogenesis-Related protein 1 (PR1) accumulation in *Arabidopsis* rosette leaves of WT, *prr2*.*1* and OE-PRR2s lines infected with *Pst* DC3000 (2.10^8^ cfu.mL^−1^) (Fig. 4E). PR1 is an antimicrobial protein considered as a key marker of defence responses associated to SA^28, 29^. Immunoblots with anti-PR1 antibody revealed that PR1 protein accumulates 24 h post-infection in the WT (Col) (Fig. 4E- lanes WT). In the same experimental conditions, PR1 was not detected at all in the *prr2* mutant, (Fig. 4E – lanes *prr2*.*1*). In contrast, PR1 production is detected from 12 hpi in OE PRR2.1 lines (*i*.*e*. earlier than in WT plants) with a significant enhanced PR1 accumulation compared to WT plants 24 hpi (Fig. 4E – lanes *OE-PRR2*.*1*). This result confirms that PRR2 positively regulates SA-dependent defence responses upon infection with *P*. *syringae*.

### PRR2 is involved in salicylic acid production and/or accumulation in response to *P*. *syringae*

**Figure 5:**
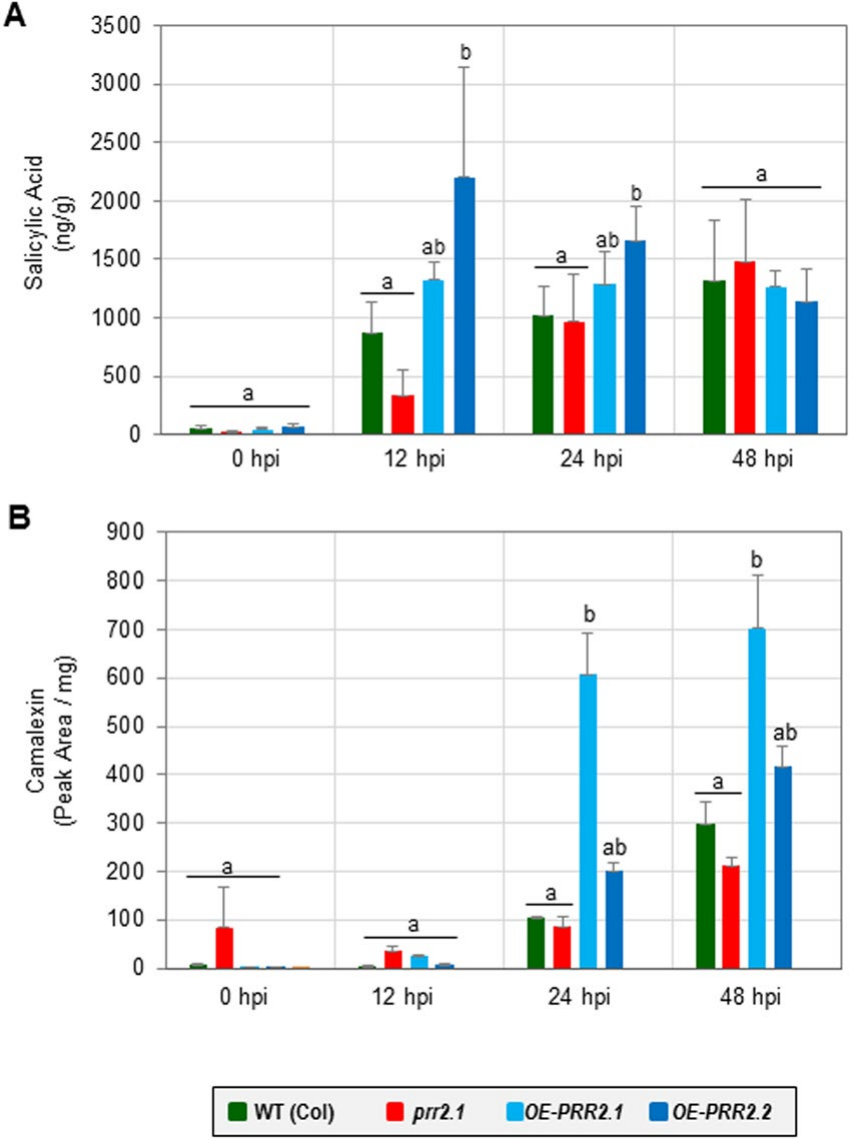
Quantification of total SA and camalexin content in *prr2* genotypes in response to *Pseudomonas syringae* inoculation. (**A**) Quantification of SA levels in leaves of 4-week-old plants of *Arabidopsis thaliana* in control conditions or after inoculation with *Pst* DC3000. SA quantifications were performed at 0 h, 12 h, 24 h and 48 h post-inoculation in WT (Col) and *prr2* plants (*prr2*.*1*, *OE-PRR2*.*1* and *OE-PRR2*.*2*) with *Pst* DC3000 at 10^7^ cfu.mL^−1^. (**B**) Quantification of camalexin levels in leaves of 4-week-old plants of *Arabidopsis thaliana* in control conditions or after inoculation with *Pst* DC3000. Camalexin content was measured in WT, *prr2* and OE-PRR2 lines after inoculation with *Pst* DC3000. For **A** and **B**, each bar represents the mean and standard error of three biological replicates. Bars sharing the same letter are not significantly different according to Dunnett’s test (p-value < 0.05).

Data clearly showed that *PRR2* gene expression is SA-dependent and that reciprocally, knocking-down or over-expressing *PRR2* alters the expression of SA-associated defence genes and PR1 protein accumulation in the context of *P*. *syringae* infection. In *Arabidopsis*, an accumulation of SA is essential to the activation of SA-dependent responses and complete resistance against *P*. *syringae* ^23^. Hence, we determined whether SA level is altered in *prr2* lines. Total SA quantification was performed in leaf tissues under control conditions and at different time-points after inoculation with *Pst* DC3000 (Fig. 5A). As already reported following *Pst* infection, SA content increased by about 16-fold in inoculated leaves of WT plants (Col) after 12 hpi compared to T0 (Fig. 5A). SA level remains elevated in the WT at 24 hpi (∼20-fold) and 48 hpi (25-fold). Compared to WT plants, no significant change was quantified in *prr2* mutant, although SA content appears to be slightly but not significantly reduced after 12 hpi. On the contrary, the level of SA concentration is transiently higher (1.5 to 2.5-fold) in *OE-PRR2*.*1* and *OE-PRR2*.*2* lines compared to the WT after 12 hpi. After 48 h, SA levels are not significantly different between WT and *PRR2* over-expressing lines.

According to these results, we propose that PRR2 contributes to a transient accumulation of SA in response to *Pst* DC3000. This leads to enhanced defence responses associated to SA and increased disease resistance against *P*. *syringae*.

### PRR2 modifies camalexin content in response to Pseudomonas syringae pv tomato infection

Camalexin, one of the major phytoalexin produced by *Arabidopsis thaliana* is essential for resistance to fungal plant pathogens but also to bacteria such as *Pseudomonas syringae* ^30^. Camalexin is produced from tryptophan through the activities of several enzymes^31^. Those include the cytochrome P450 monooxygenase CYP71B15/PAD3 whose expression appears to be altered in *prr2* genotypes (Fig. 4D). To evaluate the role of PRR2 in camalexin accumulation (Fig. 5B), we measured camalexin content in *prr2* mutants and *OE-PRR2s* transgenic lines in response to *Pst* infection and results were compared to those obtained in WT plants (Fig. 5B). Following *Pst* infection, camalexin levels in WT increases by about 20-fold and 60-fold at 24 hpi and 48 hpi, respectively. Interestingly, a significantly higher increase is observed in OE lines with up to 6-fold more camalexin at 24 hpi (Fig. 5B). A reduced but not significant camalexin content was quantified in *prr2*.*1* mutant compared to the WT 48 hpi. No significant difference was observed for other time-points. These results correlate with the expression profile of genes involved in camalexin production in *prr2* lines and confirm the function of PRR2 as a positive regulator of camalexin production.

## Discussion

Since *A*. *thaliana* genome initiative and its annotation releases, the gene AT4G18020 (*PRR2*) has been described as a Pseudo-Response Regulator that belongs to a small family of plant specific transcription factors^32^. This classification is based on the occurrence of a (pseudo-) receiver domain similar to the receiver domain encountered in Authentic Response Regulators (ARRs) involved in plant hormone signal transduction^32, 33^. Despite their structural similarity with ARRs, PRRs lack essential residues required for the phospho-accepting activity in the receiver domain and exhibit other motifs and/or domains^34^. Thus in *A*. *thaliana*, 9 PRRs have been identified and ranged into two groups according to the occurrence or not of a CCT motif (first characterized in CONSTANS, a key regulator of plant flowering) in the carboxy-terminal end of the protein^35^. PRRs belonging to this group (PRR9, PRR7, PRR5, PRR3 and PRR1/TOC1) are involved in circadian rhythm and some of them have been proposed to regulate the circadian clock system by repressing clock-associated genes expression^36, 37^. The available data concerning the second group of PRRs is even more scattered. PRR2, PRR4 and PRR6 that belong to this group are characterized by the presence of a MYB related DNA-binding domain^34^. Interestingly, PRR2 presents the common features of PRRs and a conserved GCT box only encountered in GLKs (Golden2-Like) proteins that are plant specific transcription factors involved in chloroplast biogenesis^38, 39^. According to bioinformatic analyses, PRR2 relatives are only found in plants and more specifically in dicotyledonous. Until recently, the role of this PRR still remains unknown. Pan *et al*.^21^ showed that SlPRR2 influences fruit pigmentation and ripening in tomato. The *SlPRR2* overexpressing lines accumulate more carotenoid than WT in tomato fruit and possess plastids with enhanced size and chlorophyll content^21^.

We previously identified PRR2 as an interacting partner of the calcium sensor CML9^18^ which is involved in plant immunity in response to *P*. *syringae* ^17^. Therefore, we evaluated the contribution of PRR2 in plant defence responses against this bacterial pathogen. Gene expression analysis indicates that *PRR2* is expressed in all aerial organs of *Arabidopsis* adult plants and up-regulated in response to the infection with *P*. *syringae*. Our data also show that the modulation of *PRR2* expression lead to changes in the level of susceptibility to *Pseudomonas syringae*. This suggests that PRR2 counteract the effect of virulence factors and is likely to regulate plant resistance processes.

SA biosynthesis and signalling have been demonstrated to be critical for resistance against *Pst* ^40–42^. PRR2 exhibits features associated to TFs, we therefore expected that an overexpression of this gene would lead to alteration in gene expression. We indeed observed significant changes in the transcription of marker genes associated to SA metabolism and signalling. *PRR2* over-expressing lines exhibit early and enhanced expression of these genes compared to WT following *Pst* infection (Fig. 4). These modifications mainly affect genes involved in SA production (*CBP60g*)^43^ or in SA signalling (*WRKY6*)^44^. The PR1 protein, a classical SA-associated marker^28^, is detected earlier and at higher level in the *OE-PRR2* lines upon infection (Fig. 4E). These data are consistent with SA quantification showing a rapid and enhanced production of SA in *OE-PRR2* lines compared to WT plants following *Pseudomonas* inoculation (Fig. 5A). Together, these data indicate that PRR2 potentiates plant defence responses by regulating SA homeostasis. The regulation of SA accumulation/signalling by a CML-binding TF increases the complexity of the regulation of host immune responses. Indeed, several CaM-binding proteins act as transcriptional regulators and participate in plant immunity by regulating SA-associated responses. Among them, CAMTA3 acts as a negative regulator of SA-associated defences^11, 12^ whereas CBP60g positively contributes to SA accumulation in a pathogenic context^13, 45^. We also reported that PRR2 significantly enhances the transcription of genes encoding for enzymes involved in the production of camalexin (*MYB51*, *PAD3*). These expression patterns are consistent with the quantification of camalexin showing that *PRR2* contributes to the accumulation of this metabolite in response to *P*. *syringae* (Fig. 5B). Camalexin was previously described to be produced by *Arabidopsis* in response to the infection with bacterial pathogens and to disrupt the integrity of bacterial membranes^31, 46^. Regarding the enhanced camalexin accumulation in *OE-PRR2* lines, it would be of particular interest to test the susceptibility of these transgenic lines to other pathogens such as fungi or oomycetes^46^.

The present study and data published by Pan *et al*.^21^ support the hypothesis that PRR2 plays a dual role in plant physiology both in plant development and in response to pathogens as previously described for GLKs^38, 47^. So far, the mechanisms by which PRR2 respond to developmental and environmental stimuli in both tomato and *Arabidopsis* are unknown. We cannot rule out that these responses share common features related to chloroplast function since SA and camalexin productions are initiated in these organelles known to be key elements in plant stress responses^48, 49^. Moreover, de Torres-Zabala *et al*. recently showed that chloroplast is a key component of early immune responses in the *A*. *thaliana* – *Pseudomonas* pathosystem^50^.

Analyses of the *Arabidopsis* interactome bring new data indicating that plant TFs might function in a combinatorial fashion^5^ and according to these data, PRR2 might interact with TCP19^51^. TCPs are involved in various developmental pathways^52^ and more recently certain have been associated with plant immunity^53^. Interestingly, according to Mukhtar *et al*.^54^ and Weßling *et al*.^55^, four TCPs including TCP19 have been found to be directly targeted by effectors from *Pst* and other pathogens^54, 55^. This information suggests that a subset of TCP proteins interacting with PRR2 can modulate plant defence and/or susceptibility responses regulating both plant development and immunity. We hypothesize that PRR2 could affect plant defence response and/or susceptibility by acting as cofactor in transcriptional complexes that might also recruit calcium sensors such as CMLs for example. Activation of such complex transcription regulatory networks is transient and not sustained over the time course of an infection. This would explain why PRR2 expression level (*i*.*e*. *prr2*.*1* vs *OE-PRR2*) rather modulates the timing and amplitude of defense responses than their sustainability. It is also worth considering the fact that PRR2 interacts with CML9 in the plant nucleus^18^. We previously showed that CML9 acts as a negative regulator in the flagellin signalling pathway leading to plant defence processes^17^. In this work, we also showed that CML9 acts as a positive regulator of the plant defence against virulent bacteria (*Pst* DC3000). These observations led us to hypothesize that this complicated role of CML9 in plant immunity could be conceived by its repertoire of target proteins^17^. In this hypothesis, we can imagine that the PRR2-CML9 complex could contribute to limit the effector-triggered susceptibility. How the activity of PRR2 is regulated by Ca^2+^/CML9 and the biological relevance of this interaction is yet to be determined and will considerably increase our understanding of the contribution of Ca^2+^ signalling in modulating SA-dependent defence responses.

Thus, the next challenge will be to better understand the physiological relevance of the PRR2-CML9 interaction, taking into account the redundancy between CMLs since it has been shown that PRR2 can also interact with other CMLs (CML8 and CML11) the closest relatives of CML9^18^. Interestingly, we recently have demonstrated that CML8 is also involved in plant immunity as a positive regulator of defence responses associated to SA^56^. Finally, forthcoming work will rely on a strategy devoted to the identification of target genes of this transcription factor in order to better decipher the biological relevance of PRR2.

## Methods

### Plant materials, growth conditions and hormone treatments

Seeds from *Arabidopsis thaliana* accession Columbia (Col), T-DNA insertion line (*prr2-1*) from the GABI-Kat mutant collection (http://www.gabi-kat.de) and mutant lines impaired in salicylic acid (*sid1*, *sid2*, *nahG*), jasmonic acid (*jar1*) and PAMP perception (*fls2* and *efr*) were purchased from the Nottingham *Arabidopsis* Stock Center (http://arabidopsis.info). The WS accession and the *prr2-2* mutant line were obtained from INRA (http://www.international.inra.fr). T-DNA insertions occurred at a single locus in the *prr2-2* line and at several loci in the *prr2-1* line. Detection and transcripts quantification of *PRR2* were performed in *prr2-1* and *prr2-2* mutants using both RT-PCR and quantitative RT-PCR, and expression level of *Actin8* was used as a quantifying control (see Supplemental Informations 1 and 2).

Seeds, seedlings and adult plants were used for experiments described in this work. To avoid variations in seed quality, all the plants were grown in identical conditions at the same period and seeds were harvested and stored in the same way. To obtain seedlings, seeds were surface-sterilized and sown on agar plates containing 0.8% Murashige and Skoog (MS) medium. The plates were incubated for 3 days at 4 °C to break any residual dormancy of seeds, and then transferred into a growth chamber at 20–22 °C with a 16 h photoperiod. Exogenous SA application (50 µM) was sprayed on 2-week-old seedlings cultivated on solid MS medium and results were compared to H_2_O (mock treatment). To obtain adult plants, *Arabidopsis* plants were grown in pots filled with TKS2 peat Floratorf under growth chamber conditions at 20 °C with 16/8 h light/dark photoperiod given by fluorescent tubes 36 W (12 W m^−2^) and 60% humidity.

### Generation of *PRR2* over-expressing transgenic lines

Transgenic Arabidopsis (Col) plants were transformed with *p35S*::*cdsPRR2-HA* fused to 3HA epitope. The construct was obtained from the full length *PRR2* cDNA amplified by PCR and cloned into the pAM-PAT Gateway vector before plant transformation^57^. Two homozygous independent lines (*OE-PRR2*.*1* and *OE-PRR2*.*2*) were obtained and characterized to check for the presence of the transcript by quantitative RT-PCR with specific primers (Table S1). The 3HA-tagged PRR2 protein was detected by western blot using an antibody directed against the 3HA epitope on total protein extracts (Supplemental Information 2).

### *PRR2* promoter-*uidA* reporter gene expression in transgenic plants

To generate the *PRR2* *promoter*::*uidA* construct, the 5′ flanking DNA region of the *PRR2* coding sequence was PCR-amplified using the primers described in table S1 (Supplemental Data) to obtain a DNA fragment of 2.3 kb in size ranging from the initiation ATG codon to the upstream region corresponding to *PRR2* promoter sequence. This fragment was cloned into the destination vector pMDC162^58^ to create *Pro*_*PRR2*_::*uidA*. After DNA sequencing, the resulting plasmid was introduced into *Agrobacterium tumefaciens* (strain C58C1 (*pMP90*)), and used for transformation of *A*. *thaliana* accession Col by floral dipping^57^. Transgenic lines were selected by sowing seeds on selective medium and progeny fully resistant to hygromycin were selected and used for further experiments. For GUS staining, various tissues from T_3_ transgenic reporter lines were treated as reported by Magnan *et al*.^16^. Plant samples were then cleared of chlorophyll in ethanol and photographs of histochemical localizations of GUS activity were taken using a digital camera either under the microscope (Zeiss) or stereo-microscopy (Leica).

### Gene expression analyses

These analyses were performed as previously described by Leba *et al*.^17^. RNA extraction from leaves and reverse transcription were conducted as recommended by the manufacturers’ protocols (respectively with the Nucleospin RNA plant kit from Macherey-Nagel and the superscript reverse transcriptase II from Invitrogen). Quantitative PCR was run on a Roche lightcycler system (Roche Diagnostics) using specific pairs of primers (listed in Table S1). Each value obtained is an average of three independent biological replicates, and the experiment was repeated two times for each biological repeat. The measurements obtained for the reference gene, *actin8*, were used for data standardization. Expression analyses of defence-induced marker genes (Table S1) were performed by quantitative PCR with Fluidigm Biomark^®^ technology (Genomic GenoToul). First-strand cDNA templates were pre-amplified with TaqMan preamp master mix and reactions were achieved in a Fluidigm Biomark® BMK-M-96.96 plate according to the manufacturer’s recommendations. Relative gene expression values were determined using the 2^−∆∆CT^ method from Livak and Schmittgen^59^. The expression analyses data are an average of four independent replicates. As described before, the *actin8* gene expression levels were used for data standardization. In all these experiments, the wild-type plant (Col) was used as the reference.

### Plant inoculations and in planta bacterial growth determination

*Pseudomonas syringae* strains used in this study were grown at 28 °C on LB medium supplemented with the appropriate antibiotics: 50 μg/mL of rifampicin (*Pst* DC3000 and *Ps pv maculicola*). To homogenize and enhance infection efficiency, the plants were placed in high humidity atmosphere 12 h before infection with bacterial inoculum prepared at the indicated densities and sprayed directly on leaf surfaces. Quantification of *in planta* bacterial growth was performed as previously described^60^.

### Immunoblot assays and detection of PR1 protein

Three-week-old plants sprayed with *Pseudomonas syringae DC3000* (2.10^8^ cfu.mL^−1^) were harvested and total protein extraction was performed by tissue homogenization in extraction buffer (50 mM HEPES NaOH (pH7,5), 1% (v/v) plant anti protease (Sigma P9599), 5 mM NaF, 50 mM K_4_P_2_O_7_, 10 mM Na_3_VO_4_, 10 mM MgCl2, 1 mM DTT, 2 µM Leupeptine, 100 µM PMSF, 50 µM MG132). Proteins were separated on a 15% SDS-polyacrylamide gel and were detected by immunoblot analyses using either anti-PR1 (AGRISERA) or anti-GAPDH (as loading control) (COVALAB) antibodies.

### Salicylic acid and camalexin quantification in *Arabidopsis* leaves

SA and camalexin determinations were performed on three biological replicates consisting of a minimum of five infected or mock-treated leaves from five different plants. Samples were collected at indicated times, frozen immediately in liquid nitrogen. Tissues were then ground to a powder and hormone extraction was performed as described by Vadassery *et al*.^61^ with an internal standard D4-SA added in the extraction buffer (Santa Cruz Biotechnology). The homogenate was mixed for 30 min and centrifuged at 14,000 rpm for 20 min at 4 °C. After the supernatant was collected, the homogenate was re-extracted with 500 µL of methanol, mixed, and centrifuged, and supernatants were pooled. The combined extracts were evaporated in a SpeedVac at 30 °C and re-dissolved in 500 µL of methanol. Chromatography was performed on an Agilent 1200 HPLC system (Agilent Technologies). Separation was achieved on a Zorbax Eclipse XDB-C18 column (50 × 4.6mm, 1.8 µm; Agilent). Formic acid (0.05%) in water and acetonitrile were employed as mobile phases A and B, respectively. The elution profile was as follows: 0 to 0.5 min, 5% B; 0.5 to 9.5 min, 5% to 42% B; 9.5 to 9.51 min, 42% to 100% B; 9.51 to 12 min, 100% B; and 12.1 to 15 min, 5% B. The mobile phase flow rate was 1.1 mL.min−1. The column temperature was maintained at 25 °C. An API 3200 tandem mass spectrometer (Applied Biosystems) equipped with a turbospray ion source was operated in the negative ionization mode. The instrument parameters were optimized by infusion experiments with pure standards, where available. The ion spray voltage was maintained at −4,500 eV. The turbo gas temperature was set at 700 °C. Nebulizing gas was set at 60 ψ, curtain gas at 25 ψ, heating gas at 60 ψ, and collision gas at 7 ψ. Multiple reaction monitoring was used to monitor analyte parent ion → product ion: mass-to-charge ratio [*m/z*] 136.9 → 93.0 (collision energy [CE], −22 V; declustering potential [DP], −35 V) for salicylic acid; m/z 140.9 → 97.0 (CE, −22 V; DP, −35 V) for D4-salicylic acid. Both Q1 and Q3 quadrupoles were maintained at unit resolution. Analyst 1.5 software (Applied Biosystems) was used for data acquisition and processing. Linearity in ionization efficiencies was verified by analyzing dilution series of standard mixtures. SA was quantified relative to the signal of their corresponding internal standard.

Camalexin was analyzed from the same extract as salicylic acid (see above). Chromatography was performed on an Agilent 1200 HPLC system (Agilent Technologies, Boeblingen, Germany). Separation was achieved on a Zorbax Eclipse XDB-C18 column (50 × 4.6 mm, 1.8 µm, Agilent, Germany). Formic acid (0.05%) in water and acetonitrile were employed as mobile phases A and B respectively. The elution profile was: 0–0.5 min, 5%B; 0.5–1 min, 5–100% B in A; 1–2 min 100% B and 2.1–4. 5 min 5% B. The mobile phase flow rate was 0.8 ml/min. The column temperature was maintained at 25 °C. An API 3200 tandem mass spectrometer (Applied Biosystems, Darmstadt, Germany) equipped with a turbospray ion source was operated in positive ionization mode. The ionspray voltage was maintained at 5500 V. The turbo gas temperature was set at 700 °C. Nebulizing gas was set at 70 psi, curtain gas at 35 psi, heating gas at 70 psi and collision gas at 2 psi. Multiple reaction monitoring (MRM) was used to monitor analyte parent ion → product ion: *m/z* 201.09 → 59.01 (collision energy (CE) 45 V; declustering potential (DP) 51 V). Both Q1 and Q3 quadrupoles were maintained at unit resolution. Analyst 1.5 software (Applied Biosystems, Darmstadt, Germany) was used for data acquisition and processing. Data are expressed as peak area of the LC-MS/MS trace per mg plant weight.

### Statistical analysis

All statistical analyses were performed using the software Statgraphics Centurion XV (SigmaPlus, France).

### Accession numbers

PRR2 (AT4G18020); Actin8 (AT1G49240); WRKY6 (AT1G62300); MYB51 (AT1G18570); CYP71B15/PAD3 (AT3G26830), CBP60G (AT5G26920).

## Electronic supplementary material

Supplementary Information
