# Supplementary material for: PRR2, a pseudo-response regulator, promotes salicylic acid and camalexin accumulation during plant immunity
Source: Sci Rep. 2017 Aug 1;7:6979. doi: 10.1038/s41598-017-07535-8 (PMC5539105; doi:10.1038/s41598-017-07535-8)
Supplement: Supplementary file 1 — Supplementary Information [file 41598_2017_7535_MOESM1_ESM.pdf]

Supplemental Informations

PRR2, a pseudo-response regulator, promotes salicylic acid and camalexin accumulation during plant immunity

Cheval C.[ɣ1,4]**, Perez M.**[ɣ 1]**, Leba L.J.**[1,5]**, Ranty B.**[1]**, Perochon A.**[1,6]**, Reichelt M.**[2]**, Mithöfer A.**[3]**, Robe E.**[1]**, Mazars C.**[1]**, Galaud J.P.**[1]**, Aldon D.**[1*]

Legends of Supplemental Informations

Supplemental Information 1

Molecular characterization of *prr2* mutant lines. *The prr2* mutants were identified in the *Arabidopsis* T-DNA insertion lines collections from plant resource centers. (**A**) Schematic illustration of the T-DNA insertions into the genomic region of *PRR2* gene. The *prr2-1* (Col) and *prr2-2* (WS) mutants contain a T-DNA insertion in the first intron, 629bp and 425bp downstream of the transcription start site of *PRR2* gene respectively. (**B**) Semi-quantitative analysis of *PRR2* transcripts accumulation was carried out on homozygous lines using primers *F1* and *R1* (see panel A for primers positions). Actin 2 was used as a control. Data indicate that *PRR2* transcripts can be detected in both insertional mutants, but they accumulate at a lower level than that observed in wild-types, indicating a down-regulation of *PRR2* expression in the mutants. (**C**) Quantitative RT-QPCR was performed to measure the relative *PRR2* gene expression level in mutants compared to WT. As shown, *PRR2* expression is respectively decreased by about 3 and 7 fold in *prr2-1* and *prr2-2* compared to Col and WS.

Supplemental Information 2

Molecular characterization of Over-Expressing *PRR2* transgenic lines. (**A**) Relative expression of *PRR2-3HA* transcript in *p35S::$_{CDS}$PRR2.1* (*OE-PRR2.1*) and *p35S::$_{CDS}$PRR2.2* (*OE-PRR2.2*) lines compared to WT (Col) by quantitative RT-PCR. (**B**) Western blot of total protein from WT (Col), *OE-PRR2.1* and *OE-PRR2.2*. Total proteins were extracted and separated in SDS-PAGE before blotting and

immunodetection using antibodies raised against the 3HA epitope. Presence and length of the 3HA-tagged PRR2 protein is indicated by the arrow.

Supplemental Information 3

(**A**) Phenotype of 6-day-old WT (Col), *OE-PRR2.1*, *OE-PRR2.2, prr2.1* and WT (*WS) and prr2.2* plants grown in standard conditions. All plants were photographed from the same distance. (**B**) Phenotype of 4-week-old WT (Col0), *prr2.1*, *OE-PRR2.1*, *OE-PRR2.2* and WT (WS*) and prr2.2* plants grown in standard conditions. All plants were photographed from the same distance.

Supplemental Information 4

Quantifications of *in planta* bacterial growth in WT (WS) and *prr2.2* mutant line. Bacterial growth quantifications were performed at 0 and 1 dpi with *Pst* DC3000 **(A)** or *Pseudomonas syringae pv maculicola* **(B)**. Data are representative of 5 replicates of three independent experiments. P values were calculated using the two-tailed Mann-Whitney U-test to indicate significant differences between the genotypes. Asterisks illustrate $p<0.05$. Error bars represent SEM.

Table S1: List of primers used for quantitative PCR and molecular characterization of the *prr2* genotypes**.**

Name	Sequence	
ACTIN8	5’-CACCCGAGAGGAAGTACAGTG-3’	Forward
	5’-CATACTCTGCCTTAGAGATCCACA-3’	Reverse
PRR2	5’- GAGCGATTCCACCTTTGTT-3’	Forward
	5’- CAACCCCATGCATTACCG -3'	Reverse
WRKY6	5'- GCAACAGCAACAACAGAACAA -3'	Forward
	5'- TGCCTTGGTACTATCGTCTCC-3'	Reverse
MYB51	5'- GGCCAATTATCTTAGACCTGACA -3'	Forward
	5'- CCACGAGCTATAGCAGACCATT -3'	Reverse
CYP71B15 / PAD3	5'- CACCACTGATCATCTCAAAGGA -3'	Forward
	5'- CGGTCATTCCCCATAGTGTT -3'	Reverse
CBP60G	5'- ATCGCAGCACATCGACTTT -3'	Forward
	5'- GTGGACCGTTGAGCTTGAA -3'	Reverse
PromPRR2	5'- CATACGGTTTTTATAAGTAATGAAGC -3'	Forward
	5'- CATAGCTGGTTTTGCATCGTTTTTG -3'	Reverse

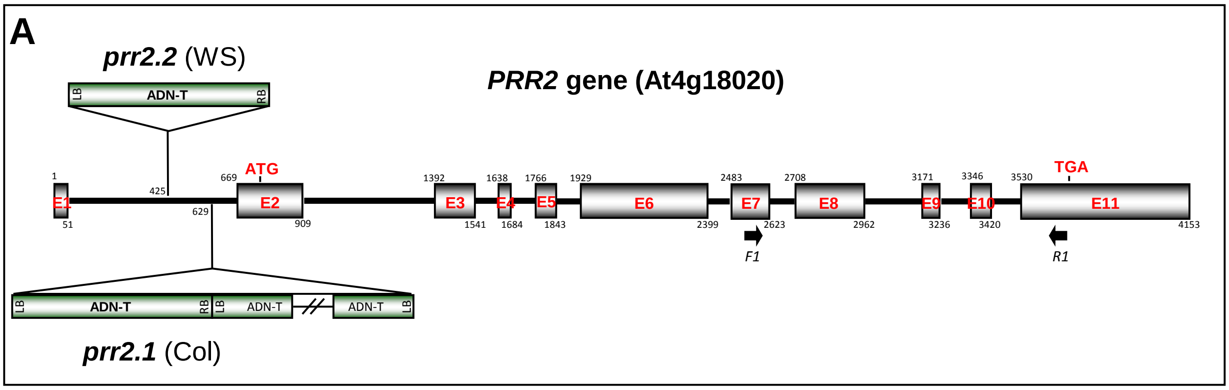
A
prr2.2 (WS)
LB ADN-T RB
PRR2 gene (At4g18020)
1
425
669 ATG
629
1392 1638 1766 1929 2483 2708 3171 3346 3530 TGA
E1 E2 E3 E4 E5 E6 E7 E8 E9 E10 E11
51 909 1541 1684 1843 2399 2623 2962 3236 3420 4153
F1 R1
LB ADN-T RB LB ADN-T ADN-T LB
prr2.1 (Col)

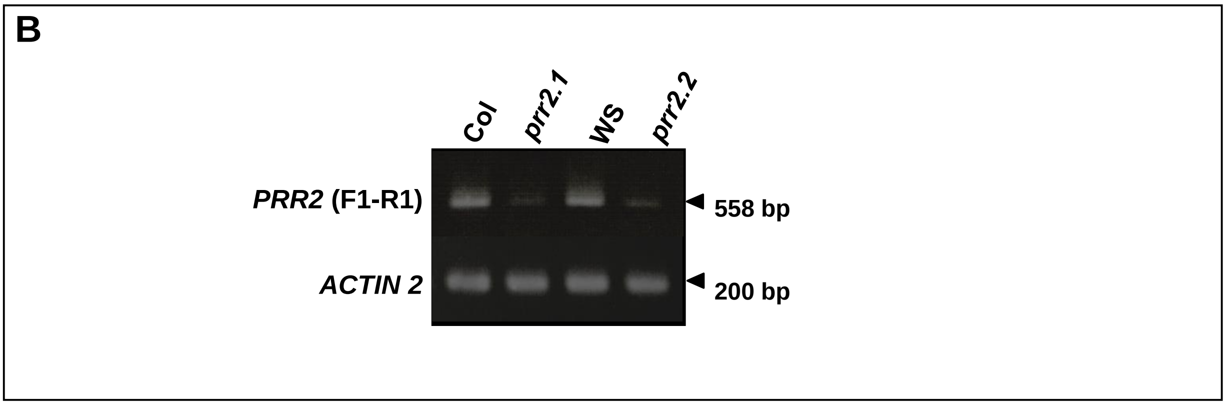
B
Col prr2.1 WS prr2.2
PRR2 (F1-R1)
ACTIN 2
558 bp
200 bp

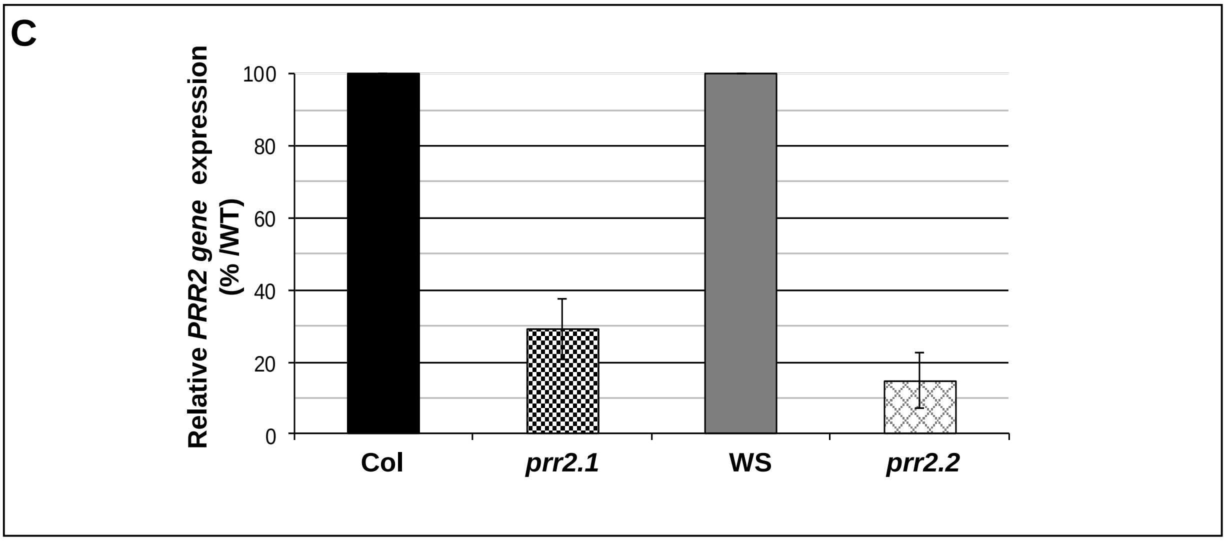
C
Relative PRR2 gene expression (% /WT)
100
80
60
40
20
0
Col prr2.1 WS prr2.2

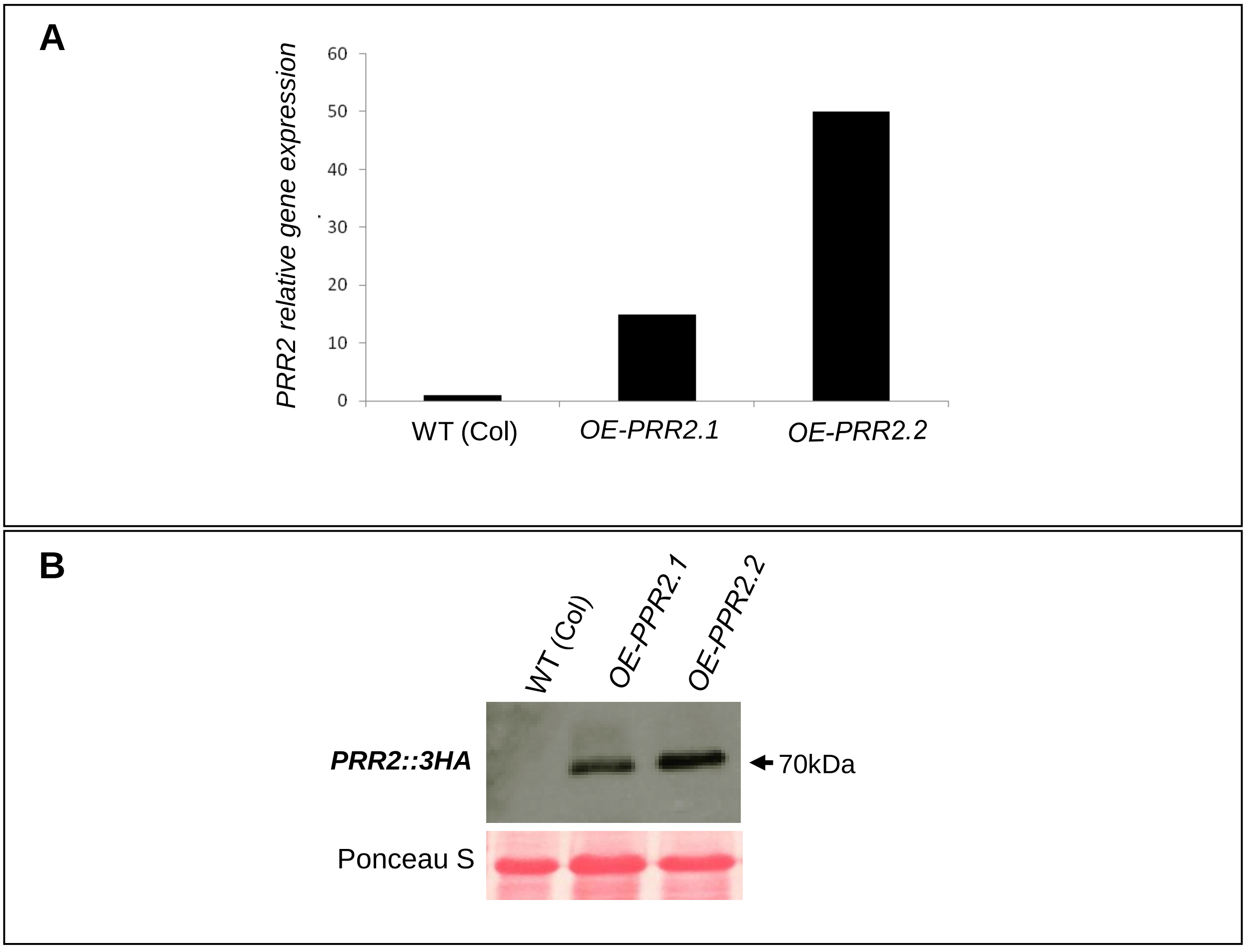
A
PRR2 relative gene expression
60
50
40
30
20
10
0
WT (Col)
OE-PRR2.1
OE-PRR2.2
B
WT (Col)
OE-PPR2.1
OE-PPR2.2
PRR2::3HA
70kDa
Ponceau S

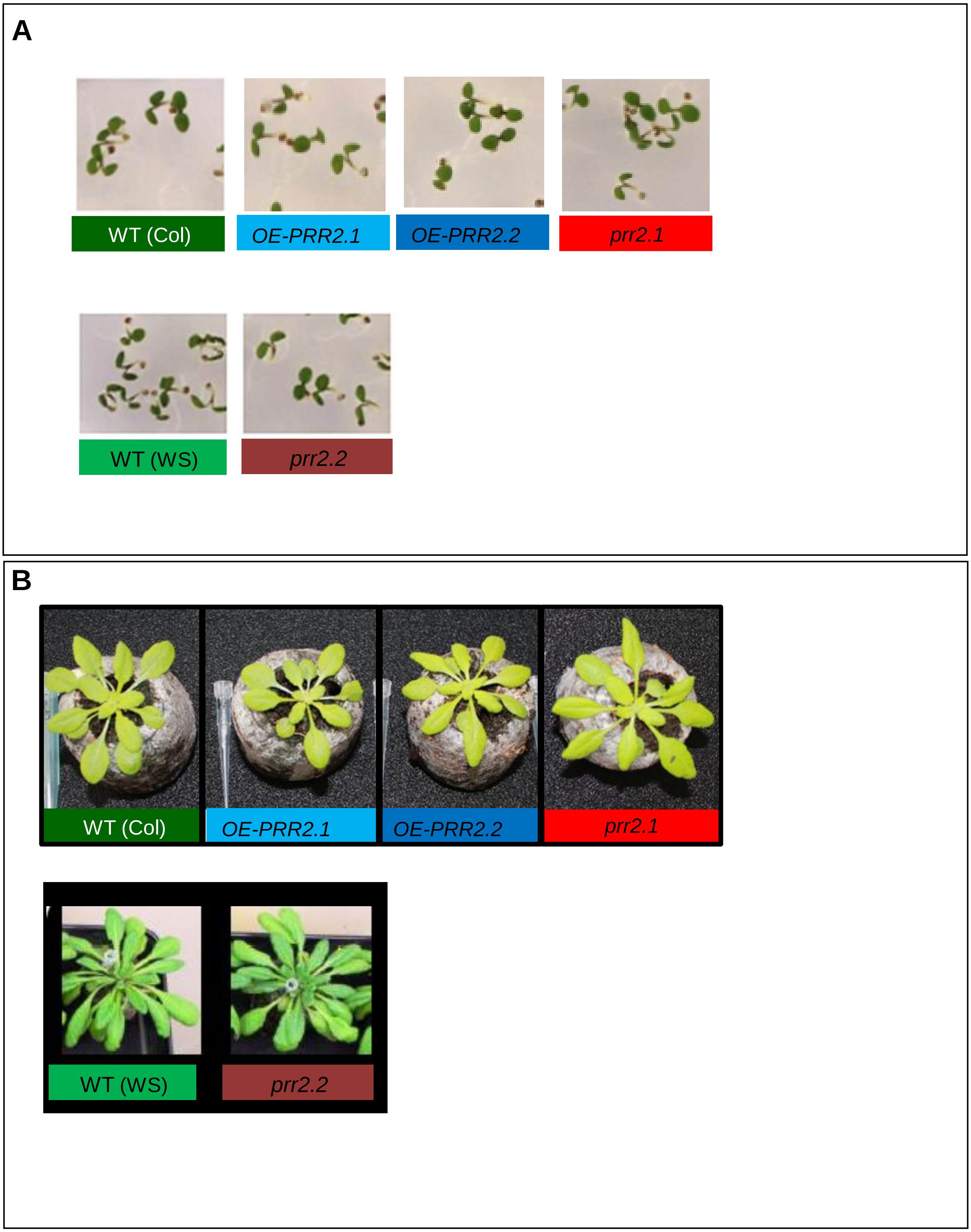
A
WT (Col)
OE-PRR2.1
OE-PRR2.2
prr2.1
WT (WS)
prr2.2
B
WT (Col)
OE-PRR2.1
OE-PRR2.2
prr2.1
WT (WS)
prr2.2

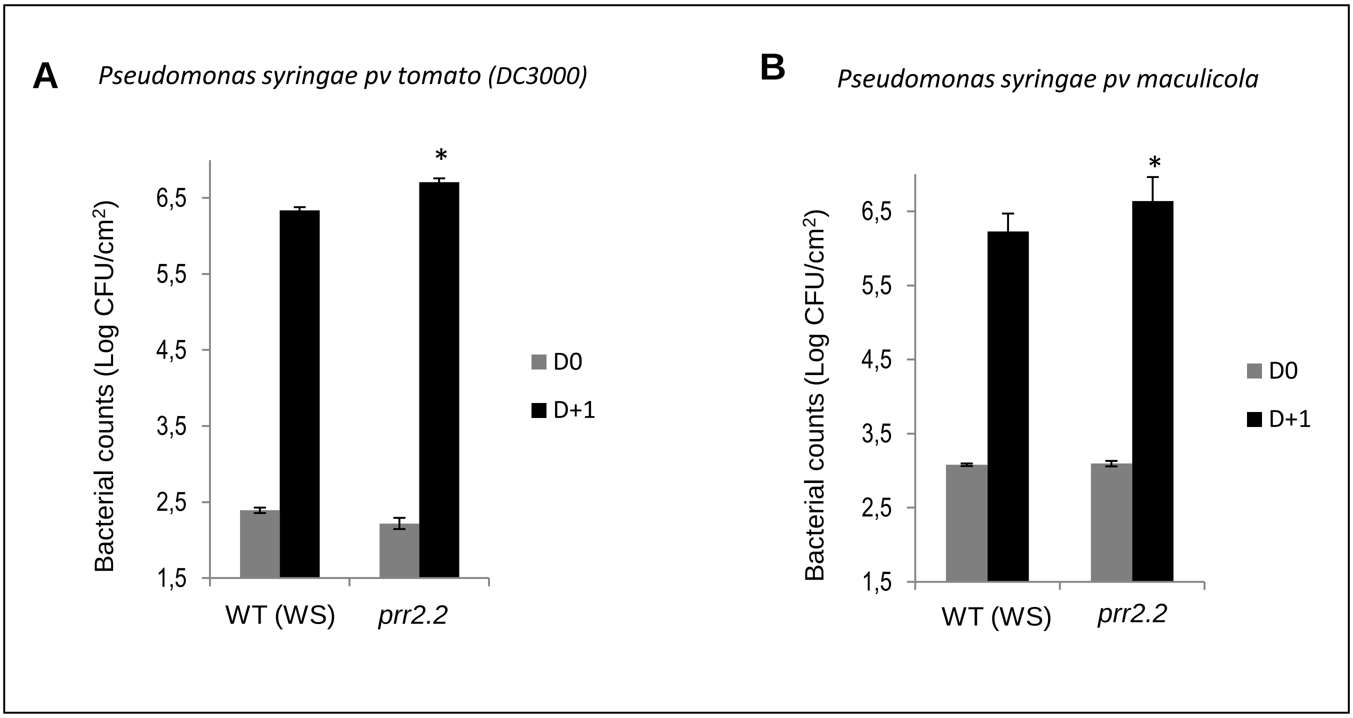
A
Pseudomonas syringae pv tomato (DC3000)
Bacterial counts (Log CFU/cm2)
6,5
5,5
4,5
3,5
2,5
1,5
*
WT (WS)
prr2.2
D0
D+1
B
Pseudomonas syringae pv maculicola
Bacterial counts (Log CFU/cm2)
6,5
5,5
4,5
3,5
2,5
1,5
*
WT (WS)
prr2.2
D0
D+1
